# Routine Blood-Based Parameters Associated with Invasive Cervical Cancer Versus High-Grade Cervical Intraepithelial Neoplasia: Development of a Retrospective Diagnostic Prediction Model

**DOI:** 10.3390/diagnostics16182884

**Published:** 2026-09-08

**Authors:** Isik Sozen, Isil Turan Bakirci, Elif Ataseven, Piril Ustundag, Yahya Ozgun Oner, Busra Ebrar Hostali, Ilkbal Temel Yuksel

**Affiliations:** 1 Department of Gynecologic Oncology, Basaksehir Cam and Sakura City Hospital, Istanbul 34480, Türkiye; yahyaozgunoner@gmail.com (Y.O.O.); drilkbaltemel@gmail.com (I.T.Y.); 2 Department of Perinatology, Basaksehir Cam and Sakura City Hospital, Istanbul 34480, Türkiye; isilturan@yahoo.com; 3 Department of Obstetrics and Gynecology, Basaksehir Cam and Sakura City Hospital, Istanbul 34480, Türkiye; dr.elifataseven@gmail.com (E.A.); pirilustundag93@gmail.com (P.U.); ebrar.hostali@gmail.com (B.E.H.)

**Keywords:** cervical cancer, cervical intraepithelial neoplasia, neutrophil-to-lymphocyte ratio, diagnostic prediction model, internal validation, tumor markers

## Abstract

**Background/Objectives:** Distinguishing invasive cervical cancer from high-grade cervical intraepithelial neoplasia (CIN) before histopathology remains difficult. We evaluated routine blood-based parameters for this distinction and developed and internally validated a parsimonious prediction model, testing its incremental value over age. **Methods:** In this retrospective diagnostic accuracy study with two-gate (case–control) sampling, we analyzed 137 unique patients with histopathologically confirmed cervical cancer and 238 with high-grade CIN after duplicate removal. A four-variable model (age, CEA, neutrophil-to-lymphocyte ratio (NLR), and hemoglobin) was developed and internally validated by bootstrap optimism correction, calibration, and decision-curve analysis, with age-matched and subgroup sensitivity analyses. **Results:** The strongest single discriminators were age (AUC 0.826), C-reactive protein (0.805), and albumin (0.772). The continuous model showed an apparent AUC of 0.859 (optimism-corrected 0.853; calibration slope 0.962; Brier 0.136). The gain over age alone was statistically significant but modest (ΔAUC +0.027; DeLong *p* = 0.015). After 1:1 age matching, age no longer discriminated (AUC 0.49), whereas the model retained moderate discrimination (AUC 0.686), with NLR independent. Performance was lower for CIN 3 versus early-stage cancer (AUC 0.759). **Conclusions:** The model may provide adjunctive risk-stratification information, but clinical implementation requires external validation in prospectively assembled cohorts; the findings are hypothesis-generating.

## 1. Introduction

Despite the spread of organized HPV-based screening and vaccination, cervical cancer continues to rank among the most frequent causes of cancer death in women worldwide [1,2]. Its principal precursor, high-grade cervical intraepithelial neoplasia (CIN 2/3), is a premalignant lesion that can progress to invasive disease if it is left untreated [3]. Telling persistent high-grade CIN apart from early invasion, therefore, carries real consequences, because the answer governs how extensive surgery needs to be, whether adjuvant treatment is warranted, and how a patient is counseled [3]. Histopathology remains the reference standard for that distinction, yet it is invasive and not free of interobserver variation [4,5], which has long motivated a search for non-invasive markers able to inform the decision before tissue is obtained.

Systemic inflammation is now understood to contribute to tumor progression, and several inflammation-based indices derived from the complete blood count, among them the neutrophil-to-lymphocyte ratio (NLR), the platelet-to-lymphocyte ratio (PLR), and the systemic immune–inflammation index (SII), have been associated with cervical cancer stage, clinicopathologic burden, and survival outcomes [6,7,8,9]. Pretreatment anemia is frequently reported in this population, and inflammation-based composites such as the C-reactive protein-to-albumin ratio and the hemoglobin, albumin, lymphocyte, and platelet (HALP) score have likewise been linked to survival [10,11,12]. Work on CIN itself has taken a different direction, concentrating on specialized molecular markers such as circulating microRNAs and soluble neuropilin-1 that lie outside routine practice [13,14].

Most published cervical cancer studies have examined prognosis within cancer cohorts [6,7,8,9], rather than comparing invasive cancer directly with high-grade CIN, which are the two entities that clinicians most often need to tell apart. A recent report suggests that SII and SIRI may serve as rule-out biomarkers for invasive cervical carcinoma [15], but the value of a wider panel of routine parameters, the gain from combining them, and, above all, the incremental value of any such model over patient age have not been examined systematically. We, therefore, developed and internally validated a parsimonious model built from routinely available laboratory parameters and tested its discrimination formally against age alone. Because platelet count was not available in the cervical cancer cohort, the present analysis focused on routinely available non-platelet laboratory parameters and leukocyte-based inflammatory ratios.

## 2. Materials and Methods

### 2.1. Study Design and Population

This was a single-center retrospective diagnostic accuracy study with two-gate (case–control) sampling, conducted at the Department of Gynecologic Oncology, Basaksehir Cam and Sakura City Hospital, Istanbul, Türkiye, between May 2020 and December 2024. The study protocol was approved by the Basaksehir Cam and Sakura City Hospital Clinical Research Ethics Committee (decision number: 77; date: 12 March 2025). The requirement for written informed consent was waived in view of the retrospective design and the use of fully anonymized data. All procedures were performed in accordance with the principles of the Declaration of Helsinki.

Two patient groups were enrolled. The cervical cancer group consisted of consecutive adult women with a histopathologically confirmed diagnosis of invasive cervical carcinoma. All histologic types of invasive cervical carcinoma were included because the clinical decision the model addresses—distinguishing invasive disease from a high-grade precursor—arises before the histologic subtype is established. The high-grade CIN group consisted of consecutive adult women whose loop electrosurgical excision procedure (LEEP) specimens demonstrated CIN 2, CIN 3, or CIN 2-3 on final pathological evaluation. The reference standard in both groups was the histopathological diagnosis rendered during routine clinical care by the institutional pathology service; the index laboratory parameters played no role in histopathological classification. The exclusion criteria for both groups were (i) history of any other active malignancy within the preceding five years; (ii) ongoing acute or chronic infection at the time of blood sampling; (iii) having a known autoimmune disease, hematological disorder, or active hepatic or renal disease; (iv) chronic use of corticosteroids or other immunosuppressive agents; (v) pregnancy or being in the postpartum period (≤6 weeks); and (vi) the absence of baseline laboratory data obtained before any surgical or oncological intervention. After these exclusions, 159 cervical cancer and 240 high-grade CIN records were identified. Cross-checking by national identity number revealed duplicate entries of the same patient (22 excess records in the cancer cohort and 2 in the CIN cohort, including one patient entered three times). Because encounter dates were not uniformly retrievable, the record with the most complete laboratory data was retained for each patient, leaving 137 unique cervical cancer and 238 unique high-grade CIN patients for analysis (participant flow; Supplementary Figure S4). A sensitivity analysis using the first-listed record instead is reported in Section 3.5.

### 2.2. Data Collection and Laboratory Measurements

Demographic, obstetric, and clinical data were extracted from electronic medical records, including age, gravidity, parity, menopausal status, and comorbidities. For the cancer group, histological subtype, FIGO 2018 stage, lymphovascular space invasion (LVSI), and lymph node involvement were recorded; for the CIN group, the LEEP grade was recorded. Laboratory parameters were obtained from peripheral venous blood drawn at diagnosis, before any treatment, and included hemoglobin, hematocrit, white blood cell count, and absolute neutrophil, lymphocyte, and monocyte counts; fibrinogen, C-reactive protein, albumin, alkaline phosphatase, and lactate dehydrogenase; and the tumor markers CEA, CA-15-3, CA-19-9, and CA-125. The NLR (neutrophils/lymphocytes) and MLR (monocytes/lymphocytes) were derived from the complete blood count. Complete blood counts, including absolute neutrophil, lymphocyte, and monocyte counts, were measured on a Sysmex XN-1000 hematology analyzer (Sysmex Corporation, Kobe, Japan). Biochemical parameters, C-reactive protein, and the tumor markers CEA, CA-15-3, CA-19-9, and CA-125 were measured on a Roche Cobas 8000 analyzer (Roche Diagnostics, Mannheim, Germany), and fibrinogen on a Roche Cobas t 711 coagulation analyzer (Roche Diagnostics, Mannheim, Germany). All analyses were performed at the institutional central laboratory with the manufacturers’ reagents and calibrators and with internal and external quality control.

### 2.3. Statistical Analysis

All eligible consecutive patients diagnosed during the study period were included; an a priori sample size calculation and post hoc power analysis were not used to justify the sample. Instead, the precision of all estimates is conveyed through effect sizes and 95% confidence intervals. Continuous variables are reported as medians (interquartile range (IQR)) and compared with the Mann–Whitney U test; categorical variables are reported as frequencies (percentage) and compared with the Pearson chi-square or the Fisher exact test. The diagnostic performance of individual parameters was assessed by ROC analysis, with the area under the curve (AUC) and bootstrap (1000-resample) 95% confidence intervals; optimal cutoffs were derived by maximizing Youden’s J statistic. Because the number of laboratory parameters and subgroup comparisons was large, between-group analyses across the 19 parameters available in both cohorts and the within-cohort subgroup comparisons should be regarded as exploratory; between-group p-values are reported unadjusted, and their robustness was additionally assessed with the Benjamini–Hochberg false-discovery-rate procedure at q = 0.05. Recorded laboratory values were screened for biologically implausible entries; such values were retained in the primary analysis because source documents could not be re-verified, and their influence was examined in sensitivity analyses. A two-sided *p* < 0.05 was considered significant. Analyses were performed in Python 3.12.0 (Python Software Foundation, Wilmington, DE, USA) with SciPy 1.11.4, statsmodels 0.14.0, and scikit-learn 1.3.2. The study is reported in line with the STARD guideline for diagnostic accuracy studies [16], and the diagnostic prediction model is reported in line with the TRIPOD statement [17]. As deviations from these recommendations, the cutoffs of the dichotomized model were data-derived rather than prespecified, and the participant flow diagram (Supplementary Figure S4) could not incorporate counts of patients screened and excluded per clinical criterion before dataset assembly, which were not retrievable from the source records.

### 2.4. Model Development, Validation, and Sensitivity Analyses

A parsimonious predictive model combined one predictor from each of four clinically distinct domains, chosen among the parameters with significant univariable discrimination and low missingness: age (demographic), CEA (tumor marker), NLR (leukocyte-based inflammation), and hemoglobin (anemia); all four remained independently associated with cervical cancer in the joint multivariable logistic model. The model was specified both with continuous predictors and, for clinical interpretability, with predictors dichotomized at their ROC-derived cutoffs (age ≥ 44 years, CEA > 2.4 ng/mL, NLR > 2.88, and hemoglobin < 11.3 g/dL); odds ratios with 95% confidence intervals are reported. The continuous four-variable model was treated as the primary analytical model, whereas the dichotomized model is reported as a clinically interpretable approximation in the Supplementary Materials (Table S3). Because cutoffs were derived from and applied within the same dataset, the reported discrimination represents apparent performance, which was corrected by bootstrap optimism estimation (1000 resamples; Harrell’s method) for the AUC and for the calibration slope, following established recommendations for prediction model development and validation [18]. The bootstrap refitted the fixed four-variable model, and no variable selection step was repeated because none was applied algorithmically. The optimism estimate is, therefore, conditional on the fixed predictor set and does not capture optimism attributable to the judgment-based choice of the four predictors. Calibration was further summarized by the calibration intercept, a decile calibration plot, and the Brier score. The dichotomized cutoffs are data-derived and require external validation before any clinical application.

To establish whether the model added value beyond patient age, nested logistic models (age alone; age plus each marker; and the full model) were compared by the DeLong test for correlated AUCs [19] and by a bootstrap distribution of the difference in AUC (ΔAUC). The clinical net benefit of the full model relative to age alone and to the treat-all and treat-none strategies was examined by decision curve analysis [20]. To address confounding by age and menopausal status, an age- and menopause-adjusted model was fitted, and a 1:1 age-matched sensitivity analysis (nearest-neighbor matching, caliper 2 years) was performed, analyzed with ordinary logistic regression and verified with a conditional logistic model accounting for a matched-pair structure. Tests of individual coefficients were conducted using the Wald test. Prespecified subgroup analyses evaluated the model in CIN 3 versus early-stage (FIGO IA–IIA) cancer, in all high-grade CIN versus early-stage cancer (advanced-stage cancers excluded), and in premenopausal women only. For every variable, the available sample size and the proportion of missing data are reported; C-reactive protein and albumin, which had substantial missingness, were not entered into the multivariable model, and their univariable results are interpreted cautiously. Collinearity among the four predictors was assessed with pairwise correlations and variance inflation factors. The systemic inflammation response index (SIRI; neutrophils × monocytes/lymphocytes), which is computable without platelet counts, was evaluated post hoc in response to peer review, and the potential contribution of CRP and albumin was examined in a multiple-imputation sensitivity analysis (chained equations with posterior sampling over the six model variables; 20 imputations, 15 iterations each; and estimates pooled across imputations), with patients with and without CRP/albumin measurements compared to characterize the missingness mechanism. Because the observed missingness reflects selective test ordering and is plausibly not at random, the imputation result is interpreted strictly as a sensitivity analysis. An additional subgroup analysis compared CIN 3 with FIGO IA–IB cancers, and sensitivity analyses assessed the influence of the fifteen suspect extreme values (twelve CEA values > 100 ng/mL, one albumin value < 20 g/L, and two NLR values < 0.15). The model was refitted with these values retained (primary analysis), with all fifteen set to missing (complete-case *n* = 326), and with the CEA values rescaled by 1/100 on the assumption of a decimal-place entry error, while the albumin and NLR values, for which no plausible correction exists, were set to missing (*n* = 338).

## 3. Results

### 3.1. Baseline Characteristics

A total of 375 unique women were analyzed: 137 with cervical cancer and 238 with high-grade CIN. Cancer patients were significantly older (median: 51 [IQR: 45–62] vs. 38.5 [31–46] years; *p* < 0.001) and more frequently postmenopausal (53.7% vs. 14.8%; *p* < 0.001). Squamous-cell carcinoma predominated (114/134; 85.1%), and 61.8% of cancers were advanced-stage (FIGO 2b–4b). The baseline characteristics are shown in Table 1.

### 3.2. Laboratory Parameters and Missing Data

**Figure 1:**
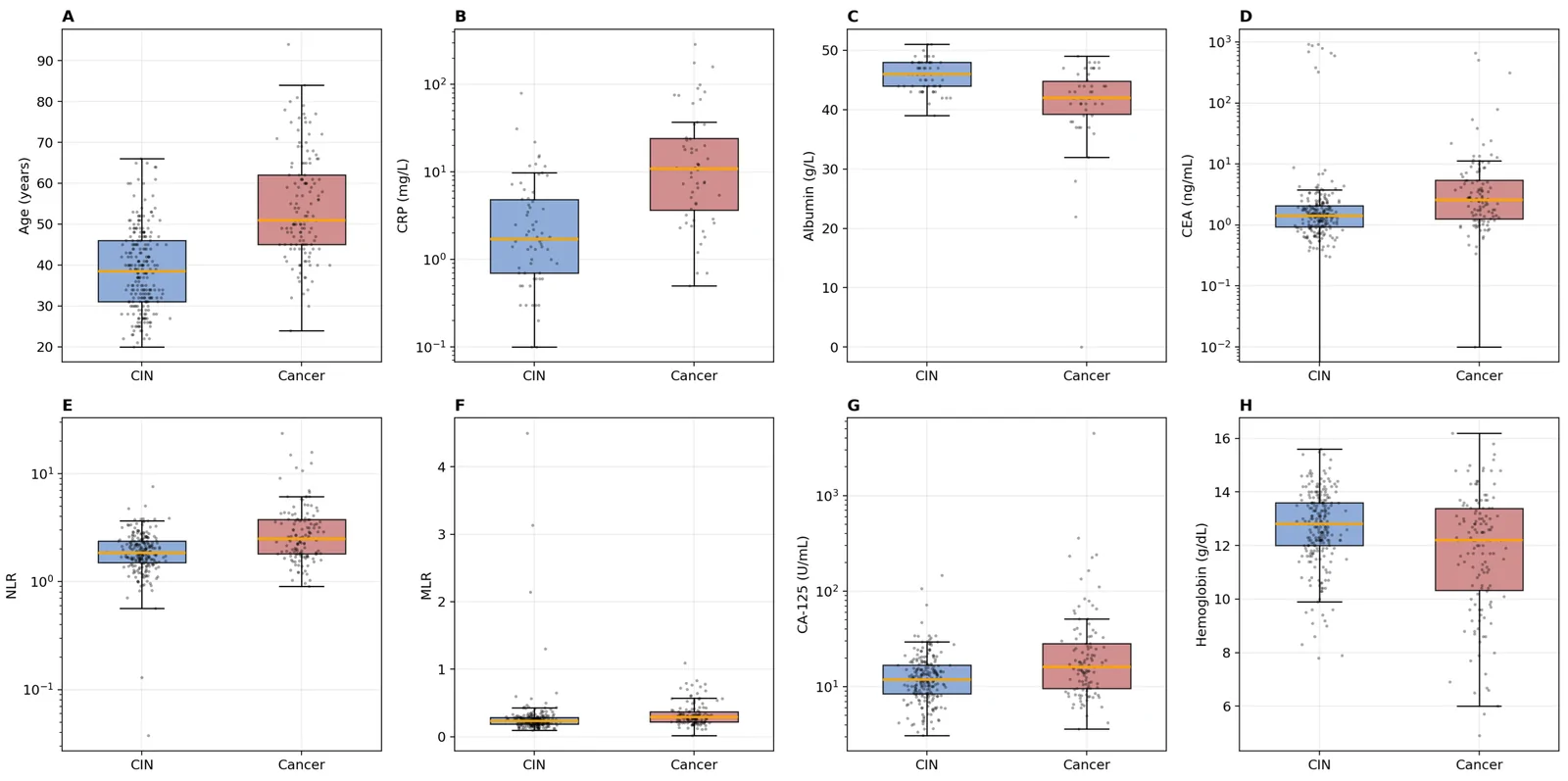
Distribution of key laboratory parameters by group ((**A**) Age (years); (**B**) CRP (mg/L) (log); (**C**) Albumin (g/L); (**D**) CEA (ng/mL) (log); (**E**) NLR (log); (**F**) MLR; (**G**) CA-125 (U/mL) (log); (**H**) Hemoglobin (g/dL)); orange line, median; points, individual patients). Skewed biomarkers (CRP, CEA, CA-125, and NLR) are shown on a logarithmic scale.

Platelet count was not recorded in the cervical cancer cohort; consequently, the platelet-derived indices PLR, SII, and AISI could not be computed for that group and were excluded from the between-group comparison. SIRI, which is leukocyte-based, was not part of the prespecified index set (NLR, MLR, and LMR) but was evaluated post hoc in response to peer review (Section 3.5). The comparison was, therefore, restricted to the 19 parameters available in both cohorts. Eighteen of the 19 comparable parameters differed significantly between groups (*p* < 0.05); monocyte count did not (*p* = 0.059). All 18 differences remained significant after Benjamini–Hochberg correction at q = 0.05. Hemoglobin, hematocrit, lymphocytes, LMR, and albumin were lower in the cancer group, whereas white blood cells, neutrophils, fibrinogen, CRP, alkaline phosphatase (ALP), LDH, CEA, CA-125, CA-15-3, CA-19-9, NLR, and MLR were higher (selected parameters are presented in Table 2; the full panel, including hematocrit, WBC, monocytes, ALP, CA-15-3, and CA-19-9, is presented in Supplementary Table S1; and the distributions of key parameters are presented in Figure 1). The availability of each key variable is summarized in Supplementary Table S2. CRP and albumin had substantial missingness (approximately 59–74%) and were, therefore, excluded from the multivariable model. The four model predictors (age, CEA, NLR, and hemoglobin) were jointly available in 339 patients; the reduction from 375 was driven mainly by missing CEA (24 cancer and 12 CIN patients). Patients excluded from the complete-case set were more often cancer patients (66.7% vs. 33.3%) and had a higher NLR (median: 2.94 vs. 1.96; *p* = 0.002), so complete-case estimates may underrepresent patients with the highest disease burden. Patients without CRP or albumin measurements were younger, less often had cancer (32.2% vs. 75.7%), and had lower CEA and NLR than patients with measurements (all *p* ≤ 0.01), indicating selective test ordering concentrated in patients with suspected invasive disease; the CRP and albumin estimates are, therefore, subject to verification bias and are interpreted cautiously. Platelet counts were absent because the cancer cohort database did not contain a platelet field, whereas the CIN database did; the unavailability reflects database construction rather than selective measurement. 

### 3.3. Single-Parameter Discrimination

Age was the single strongest discriminator (AUC = 0.826; 95% CI: 0.784–0.866), followed by CRP (0.805), albumin (0.772; lower values predicting cancer), NLR (0.690), and CEA (0.689). Among the leukocyte-based inflammatory ratios, MLR and LMR showed moderate discrimination (AUC: 0.672 each). Optimal cutoffs and operating characteristics for the leading discriminators are reported in Table 3.

### 3.4. Multivariable Model

In the primary continuous multivariable logistic model (complete-case *n* = 339), logit(P) = −5.40 + 0.123 × age (years) − 0.002 × CEA (ng/mL) + 0.495 × NLR − 0.166 × hemoglobin (g/dL), from which the predicted probability is obtained as P = 1/[1 + exp(−logit(P))]; the model achieved an AUC of 0.859 (95% CI: 0.815–0.899). In this specification, the CEA coefficient was slightly negative and non-significant (*p* = 0.12), becoming positive when the suspect extreme CEA values were excluded or rescaled (Section 3.5); modeling CEA and NLR on the logarithmic scale yielded similar discrimination (AUC: 0.854), so the untransformed specification was retained. In the clinically interpretable dichotomized approximation (ROC-derived cutoffs), all four predictors remained independently associated with cervical cancer (odds ratios: 2.50 to 8.08; coefficients, equation, and 95% CIs in Supplementary Table S3; and forest plot in Supplementary Figure S3), with an AUC of 0.831 (95% CI: 0.783–0.878), consistent with information loss from dichotomization. The four predictors showed no material collinearity (maximum absolute Spearman correlation: 0.37; variance inflation factors: 1.00–1.19).

Dichotomized model AUC: 0.831; continuous-predictor model AUC: 0.859. OR, odds ratio; CI, confidence interval. 

### 3.5. Incremental Value over Age and Internal Validation

**Figure 2:**
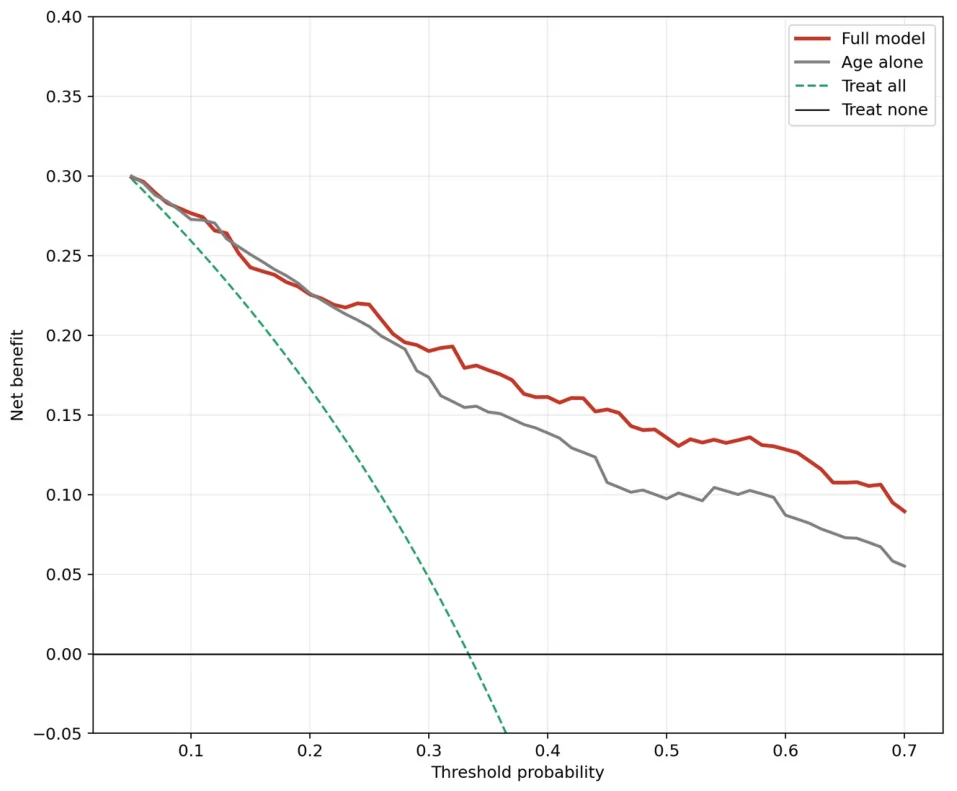
Decision curve analysis comparing the full model, age alone, and the treat-all and treat-none strategies. The full model provided similar or greater net benefit than age alone across most of the examined threshold range (approximately 0.05 to 0.70), with the advantage concentrated at threshold probabilities above approximately 0.25.

In nested continuous models, age alone yielded an AUC of 0.832; adding CEA, NLR, or hemoglobin individually produced AUCs of 0.835, 0.851, and 0.842, and the full model reached 0.859 (Table 4; Supplementary Figure S1). The slight difference between the single-parameter age AUC (0.826; Table 3) and the age-only nested-model AUC (0.832) reflects the use of the complete-case dataset for the nested model comparison. The improvement of the full model over age alone was statistically significant but modest (ΔAUC: +0.027, DeLong *p* = 0.015; bootstrap ΔAUC: +0.027, 95% CI: +0.005 to +0.048). Of the individual additions, NLR produced the largest increment (ΔAUC: +0.019), which fell just short of statistical significance (*p* = 0.053). On bootstrap internal validation, optimism was minimal (0.005), giving an optimism-corrected AUC of 0.853; the optimism-corrected calibration slope was 0.962, and the Brier score 0.136, suggesting acceptable calibration and limited optimism within the present dataset (Supplementary Figure S2). Decision curve analysis showed a small positive net benefit of the full model over age alone, concentrated at threshold probabilities above approximately 0.25 (Figure 2). The post hoc SIRI showed univariable discrimination comparable to the other leukocyte-based ratios (AUC: 0.672; 95% CI: 0.612–0.732) and did not improve the model (AUC: 0.859 with and without SIRI; Wald *p* = 0.59). In the multiple-imputation sensitivity analysis, a six-variable model additionally containing imputed CRP and albumin achieved a pooled AUC of 0.870, similar to the complete-case four-variable model. In sensitivity analyses of the fifteen suspect extreme values, model performance was stable whether the values were retained (AUC: 0.859; Brier: 0.136; *n* = 339), excluded as missing (AUC: 0.867; Brier: 0.127; *n* = 326), or corrected on the decimal-shift assumption for CEA (AUC: 0.863; Brier: 0.130; *n* = 338). The continuous CEA coefficient changed sign from slightly negative to positive when the suspect values were excluded or corrected, whereas the dichotomized CEA association was stable. Discrimination was likewise insensitive to the duplicate selection rule (first-listed record instead of the most complete record: AUC: 0.860).

### 3.6. Confounding Control and Subgroup Analyses

Age and menopausal status differed markedly between groups. In an age- and menopause-adjusted model, menopausal status was not independently associated with cancer (OR: 0.97; *p* = 0.94), whereas NLR remained independent (OR: 1.64; *p* = 0.001), and hemoglobin was not (*p* = 0.078). In a 1:1 age-matched analysis (75 pairs; median age: 48 years in both groups), age alone no longer discriminated (AUC: 0.49), whereas the four-variable model showed moderate discrimination (AUC: 0.686), with NLR remaining significant (OR: 2.03; *p* = 0.001). A conditional logistic model accounting for a matched-pair structure gave a consistent result (NLR OR: 2.07; *p* = 0.003). Across subgroups, discrimination was lower in the clinically demanding comparisons: CIN 3 versus early-stage cancer, AUC of 0.759; CIN 3 versus FIGO IA–IB cancer, 0.727; all high-grade CIN versus early-stage cancer (advanced excluded), 0.793; and premenopausal women only, 0.808 (Table 5). In these sensitivity analyses, model discrimination decreased after age matching and in the early-stage comparisons, whereas NLR remained statistically significant in the age-matched analysis.

### 3.7. Subgroup Analyses Within the Cancer Cohort

**Figure 3:**
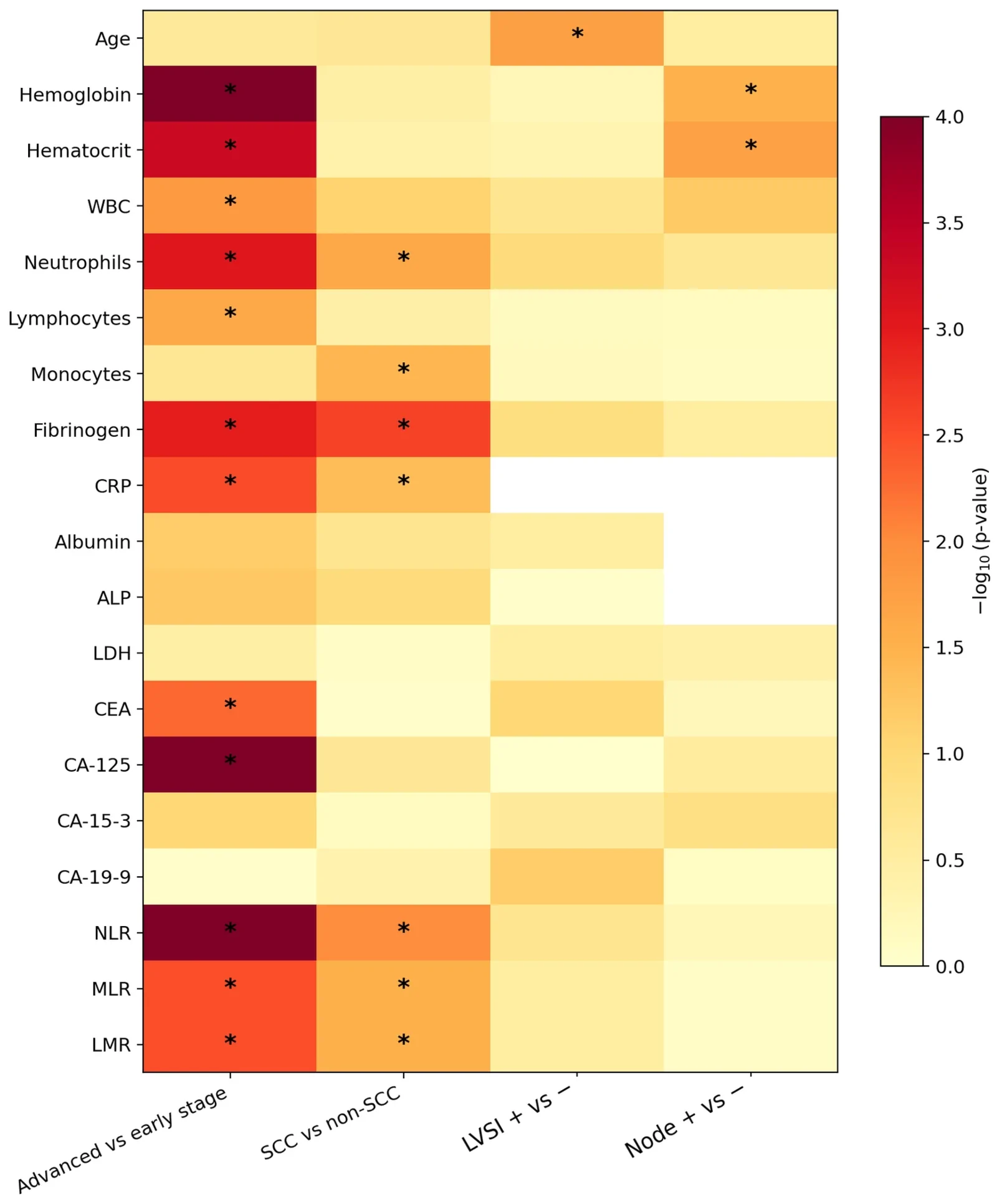
Heatmap of subgroup analyses within the cervical cancer cohort. Color intensity represents −log10 *p*-values; asterisks denote *p* < 0.05. Platelet-based indices were not available in the cancer cohort and are, therefore, not shown.

Within the cancer cohort, advanced-stage disease was associated with higher white blood cells, neutrophils, fibrinogen, CRP, CEA, CA-125, NLR, and MLR and with lower hemoglobin, hematocrit, lymphocytes, and LMR; squamous tumors showed a more pronounced leukocyte-based inflammatory profile (higher neutrophils, monocytes, fibrinogen, CRP, NLR, and MLR, and lower LMR) than non-squamous tumors; lymphovascular space invasion was associated with older age; and nodal metastasis was associated with lower hemoglobin and hematocrit. The full set of subgroup comparison *p*-values is shown in Figure 3.

## 4. Discussion

### 4.1. Principal Findings

Among 375 unique women, the primary continuous four-variable model built from age, NLR, CEA, and hemoglobin discriminated between invasive cervical cancer and high-grade CIN in this retrospective cohort, with an apparent AUC of 0.859 and an optimism-corrected AUC of 0.853, together with acceptable optimism-corrected calibration (slope: 0.962) and limited optimism. When the comparison was made formally, the gain over age alone was significant but small (ΔAUC: +0.027; DeLong *p* = 0.015), and model discrimination fell after age matching, consistent with a substantial contribution of age to discrimination. Among the evaluated laboratory markers, NLR showed the most consistent age-independent association, remaining significant after adjustment for age and menopause and within the age-matched subset.

### 4.2. Interpretation in Relation to Prior Literature

These results sit alongside a sizable literature on systemic inflammation in cervical cancer, most of which has been prognostic in nature [6,7,8,9]. Our data add a different angle, showing that NLR retains discriminatory value against a high-grade-CIN comparator and contributes the largest single-marker increment beyond age (ΔAUC: +0.019; *p* = 0.053), with the full model providing a small but statistically significant gain (*p* = 0.015). The degree of NLR elevation we observed (median: 2.48 versus 1.85) is comparable to pretreatment series such as that of Prabawa and colleagues [6], and our reading of the markers as adjuncts rather than stand-alone tests is consistent with the conclusion reached by Keszthelyi and colleagues [15]. At the same time, studies reporting only limited stand-alone discriminatory performance of NLR for cervical lesions (AUC of approximately 0.61–0.68) [21,22] caution against its use outside a multivariable context, in keeping with its modest single-marker AUC of 0.690 in the present cohort. CEA is instructive here: although its single-marker AUC was modest and it did not improve the AUC over age, it remained an independent predictor once dichotomized, a reminder that a weak univariable marker can still carry information within a multivariable model. This pattern is consistent with prior reports in which serum CEA alone showed limited discrimination for cervical cancer (single-marker AUC of 0.679 in early-stage disease) [23] and with reviews positioning CEA as an adjunct rather than a primary serum marker in cervical carcinoma [24]. Lower hemoglobin, again tracked with cancer, in keeping with the recognized association between anemia and advanced cervical disease [10] and with the prognostic signal reported for composite blood-based scores such as HALP [12].

### 4.3. Clinical Implications and Decision-Support Relevance

The subgroup analyses bear directly on clinical interpretation. In the clinically demanding comparison of CIN 3 with early-stage (FIGO IA–IIA) cancer, the AUC fell to 0.759 (0.727 when restricted to FIGO IA–IB cancers), and exclusion of advanced-stage cancers reduced discrimination, which suggests that part of the overall performance reflects advanced-stage enrichment in the cancer group rather than an ability to detect early invasion. The lower AUC in the CIN3-versus-early-stage-cancer comparison indicates that the model’s apparent overall performance should not be extrapolated to the early-invasion setting without external validation, since that is where diagnostic uncertainty is greatest. Conceptually, the model aligns with risk-based frameworks for cervical disease management [25], but it should be viewed as an adjunctive triage signal rather than a determinant of treatment selection, and not as a replacement for histopathological confirmation. Because predictive values depend on prevalence, the PPV would be substantially lower, and the NPV higher, in screening populations with a lower prevalence of invasive cancer than in this referral cohort. The decision-curve analysis is consistent with this positioning: the modest net-benefit advantage over age alone was clearest at intermediate threshold probabilities, the range relevant to prioritizing expedited diagnostic evaluation rather than definitive treatment decisions.

### 4.4. Limitations

This study has several limitations. Retrospective double entry of some patients (22 excess records in the cancer cohort and two in the CIN cohort) was identified during source data verification and corrected by retaining the most complete record per patient. A small number of biologically implausible laboratory values (twelve CEA values of 325–919 ng/mL, one albumin value of 0.06 g/L, and two NLR values below 0.15) could not be re-verified against source documents; they were retained in the primary analysis, and sensitivity analyses indicated a negligible influence on model performance. It is a single-center retrospective analysis with two-gate (case–control) sampling: the groups were assembled after histopathological diagnosis, which does not represent the prospective single-gate diagnostic moment and tends to inflate apparent performance relative to that setting. The marked age and menopause differences between groups confound the laboratory comparisons; although NLR, CEA, and hemoglobin remained associated with cancer after adjustment, age-matched discrimination was only moderate. Although CRP and albumin showed strong univariable discrimination, their high missingness (approximately 59–74%), which reflected selective test ordering (verification bias), precludes reliable inference regarding their independent contribution in the present cohort; they were excluded from the model, their univariable estimates are likely inflated, and they should not be considered candidate components based on these data. Missingness of the model predictors themselves was also differential, with cancer patients overrepresented among those excluded from the complete-case set. Squamous-cell carcinoma antigen was not recorded in either source database and could not be evaluated. Cutoffs were derived and the model evaluated within the same dataset; bootstrap optimism correction indicated limited optimism, although this estimate is conditional on the fixed predictor set and does not encompass the judgment-based selection of the four predictors, and external validation in independent prospective cohorts is required. Survival and recurrence data were unavailable, so prognostic value could not be assessed. From a PROBAST perspective, the principal potential sources of bias are the retrospective participant selection, the non-uniform missingness across predictors, the data-driven cutoffs, and the absence of external validation [26].

### 4.5. Future Validation and Implementation

Before use as a clinical decision-support tool, the model would require external validation across laboratory platforms, assessment of calibration drift over time and between institutions, and predefined procedures for model updating, alongside attention to its integration within existing clinical workflows and to the governance of any decision threshold. As an orientation for such validation, attaining a 95% confidence interval half-width of 0.05 around an assumed AUC of 0.80 would require approximately 350 patients at 40% cancer prevalence, 600 at 20%, and 1140 at 10%. Reporting in future iterations could follow the more recent extensions of the TRIPOD framework developed for regression- and machine-learning-based clinical prediction models. Finally, because psychosocial stress, neuroendocrine activation, and systemic inflammation are biologically interconnected, future prospective models could examine whether psychosocial or stress-related variables add incremental value to hematologic markers; the present dataset does not permit inference on these pathways.

## 5. Conclusions

A diagnostic prediction model combining age, NLR, CEA, and hemoglobin, all derived from a single routine blood draw, showed moderate-to-good apparent and optimism-corrected discrimination between invasive cervical cancer and high-grade CIN, with acceptable optimism-corrected calibration within the development data. Discrimination was largely driven by age; NLR provided the steadiest age-independent marker signal in this cohort, and the incremental value of the full model over age alone, while statistically significant, was modest. Performance was lower in the clinically demanding comparison of high-grade CIN with early-stage cancer. The model may provide adjunctive risk stratification information, but its clinical implementation requires external validation in prospectively assembled cohorts, ideally with formal model updating and the addition of acute-phase markers. These hypothesis-generating findings should not be extrapolated to the early invasion setting without such validation.

## Figures and Tables

**Table 1:** Demographic and clinical characteristics of the study population.

Characteristic	Cervical Cancer (*n* = 137)	High-Grade CIN (*n* = 238)	*p*
Age, median (IQR), years	51 (45–62)	38.5 (31–46)	<0.001
Age, mean ± SD, years	54.2 ± 12.9	38.9 ± 9.9	<0.001
Menopausal status, *n* (%)			<0.001
Premenopausal	63 (46.3)	201 (85.2)	
Postmenopausal	73 (53.7)	35 (14.8)	
Gravidity, median (IQR)	3 (2–5)	2 (0–3)	NT
Parity, median (IQR)	3 (2–4)	2 (0–2)	NT
Histology (cancer), *n* (%)		–	
Squamous-cell carcinoma	114 (85.1)	–	
Endocervical adenocarcinoma	19 (14.2)	–	
Adenosquamous + invasive SCC	1 (0.7)	–	
FIGO stage (cancer), *n* (%)		–	
Early (1a–2a)	50 (38.2)	–	
Advanced (2b–4b)	81 (61.8)	–	
CIN diagnosis (CIN), *n* (%)	–		
CIN 2	–	105 (44.1)	
CIN 3	–	84 (35.3)	
CIN 2–3	–	49 (20.6)	

IQR, interquartile range; SD, standard deviation; SCC, squamous-cell carcinoma; NT, not formally tested. Percentages are based on available data: histology was available for 134 cancer patients, FIGO stage for 131, and menopausal status for 136 cancer and 238 CIN patients. Continuous variables were compared with the Mann–Whitney U test; categorical variables were compared with the Pearson chi-square test. Shaded cells indicate column headers; shaded rows denote category headings, and the non-shaded rows immediately below each are the corresponding subcategories.

**Table 2:** Comparison of selected laboratory parameters between groups.

Parameter	Cancer, Median (IQR)	CIN, Median (IQR)	*p*
Hemoglobin (g/dL)	12.2 (10.3–13.4)	12.8 (12.0–13.6)	<0.001
Neutrophils (×10^3^/μL)	5.03 (3.90–6.59)	4.26 (3.51–5.41)	<0.001
Lymphocytes (×10^3^/μL)	2.08 (1.50–2.54)	2.35 (1.91–2.85)	<0.001
Fibrinogen (mg/dL)	367 (310–421)	325 (277–371)	<0.001
CRP (mg/L)	10.85 (3.65–24.18)	1.70 (0.70–4.83)	<0.001
Albumin (g/L)	42.0 (39.3–44.8)	46.0 (44.0–48.0)	<0.001
LDH (U/L)	187 (168–218)	172 (156–196)	<0.001
CEA (ng/mL)	2.58 (1.25–5.37)	1.41 (0.93–2.06)	<0.001
CA-125 (U/mL)	16.2 (9.6–28.2)	11.9 (8.5–16.9)	<0.001
NLR	2.48 (1.81–3.75)	1.85 (1.49–2.38)	<0.001
MLR	0.29 (0.22–0.37)	0.23 (0.19–0.28)	<0.001
LMR	3.48 (2.70–4.50)	4.36 (3.54–5.35)	<0.001

Selected significant parameters (Mann–Whitney U test); the full panel of 19 parameters is provided in Supplementary Table S1. Shaded cells indicate column headers. CRP, C-reactive protein; LDH, lactate dehydrogenase; CEA, carcinoembryonic antigen; NLR, neutrophil-to-lymphocyte ratio; MLR, monocyte-to-lymphocyte ratio; LMR, lymphocyte-to-monocyte ratio. Shaded cells indicate column headers.

**Table 3:** ROC analysis: leading single discriminators for cervical cancer vs. high-grade CIN.

Parameter	*n* (Ca/CIN)	AUC	95% CI	Cutoff	Sens	Spec	PPV/NPV
Age (years)	137/238	0.826	0.784–0.866	44	81.0	67.2	58.7/86.0
CRP (mg/L)	56/66	0.805	0.725–0.880	2.30	87.5	60.6	65.3/85.1
Albumin (g/L) *	50/62	0.772	0.681–0.856	42	56.0	91.9	84.8/72.2
CEA (ng/mL)	113/226	0.689	0.623–0.748	2.40	54.0	81.0	58.7/77.9
NLR	134/237	0.690	0.633–0.752	2.88	41.0	89.9	69.6/72.9

*n* (Ca/CIN), number of cancer and CIN patients with the parameter available; the CRP and albumin estimates rest on small, selected subsamples because of high missingness and should be read with caution. Shaded cells indicate column headers. * For albumin, lower values predict cancer. Sens, sensitivity; Spec, specificity; PPV/NPV, positive/negative predictive value. Cutoffs by Youden’s J; all *p* < 0.001. PPV and NPV reflect the disease prevalence within this retrospective cohort and should not be interpreted as population-level predictive values.

**Table 4:** Nested-model discrimination and comparison with age alone (continuous predictors, *n* = 339).

Model	AUC (95% CI)	ΔAUC vs. Age	DeLong *p*
Age alone	0.832 (0.788–0.874)	Reference	–
Age + CEA	0.835 (0.792–0.878)	+0.003	0.294
Age + NLR	0.851 (0.803–0.895)	+0.019	0.053
Age + hemoglobin	0.842 (0.795–0.885)	+0.010	0.228
Full model	0.859 (0.815–0.899)	+0.027	0.015

Bootstrap ΔAUC (full vs. age): +0.027 (95% CI: +0.005 to +0.048). Full model: optimism-corrected AUC: 0.853; calibration slope: 0.962; and Brier: 0.136. Shaded cells indicate column headers.

**Table 5:** Sensitivity and subgroup analyses (four-variable model).

Analysis	*n*	Model AUC (95% CI)	Age-Only AUC
1:1 age-matched (caliper 2 yr)	150	0.686 (0.603–0.764)	0.49
CIN 3 vs. early-stage cancer	128	0.759 (0.675–0.846)	0.747
CIN 3 vs. FIGO IA–IB cancer	118	0.727 (0.630–0.827)	0.716
All high-grade CIN vs. early cancer	273	0.793 (0.724–0.859)	0.779
Premenopausal women only	242	0.808 (0.741–0.867)	0.784

Model AUC refers to the four-variable model (age, CEA, NLR, and hemoglobin); Age-Only AUC is the discrimination of age alone within the same subgroup. The 1:1 age-matched analysis used nearest-neighbor matching with a caliper of 2 years. Shaded cells indicate column headers. AUC, area under the curve; CI, confidence interval.

## Data Availability

The data that support the findings of this study are available on reasonable request from the corresponding author. The data are not publicly available because they contain patient-level information that could compromise the privacy of research participants, and the ethical approval covering this study did not include patient consent for public data sharing.

## Supplementary Materials

The following supporting information can be downloaded at https://www.mdpi.com/article/10.3390/diagnostics16182884/s1: Table S1. Full panel of laboratory parameters: cervical cancer versus high-grade CIN; Table S2. Availability and missingness of key variables, by group; Table S3. Dichotomized approximation of the prediction model: independent predictors of cervical cancer (complete-case *n* = 339); Figure S1. ROC curves for nested logistic models, illustrating the incremental value of CEA, NLR, and hemoglobin over age alone; Figure S2. Calibration plot of the continuous four-variable model (decile observed vs. predicted probability; calibration-in-the-large intercept 0.00, optimism-corrected calibration slope 0.962, Brier score 0.136); Figure S3. Forest plot of independent predictors of cervical cancer (multivariable logistic regression; odds ratios with 95% confidence intervals, log scale); Figure S4. Participant flow (counts of patients screened and excluded per clinical criterion before dataset assembly were not retrievable from the source records; duplicate removal and complete-case counts are derived from the assembled datasets); File S1: TRIPOD 2015 Checklist; File S2: STARD 2015 Checklist.
