# Supplementary material for: Routine Blood-Based Parameters Associated with Invasive Cervical Cancer Versus High-Grade Cervical Intraepithelial Neoplasia: Development of a Retrospective Diagnostic Prediction Model
Source: Diagnostics (Basel). 2026 Sep 8;16(18):2884. doi: 10.3390/diagnostics16182884 (PMC13605862; doi:10.3390/diagnostics16182884)
Supplement: Supplementary file 1 [file diagnostics-16-02884-s001.zip › diagnostics-4405813 supplementary.pdf]

Supplementary Materials

Routine Blood-Based Parameters Associated with Invasive Cervical Cancer versus High-Grade Cervical Intraepithelial Neoplasia: Development of a Retrospective Diagnostic Prediction Model

Table S1. Full panel of laboratory parameters: cervical cancer versus high-grade CIN.

Parameter	Cancer, median (IQR)	CIN, median (IQR)	p	Cliff's δ	Hodges-Lehmann diff (95% CI)
Age (years)	51.00 (45.00–62.00)	38.50 (31.00–46.00)	<0.001	+0.65	+15.00 (+12.00 to +18.00)
Hemoglobin (g/dL)	12.20 (10.33–13.38)	12.80 (12.00–13.60)	<0.001	−0.23	−0.70 (−1.10 to −0.30)
Hematocrit (%)	37.45 (32.42–40.30)	38.50 (36.00–40.80)	0.004	−0.18	−1.50 (−2.60 to −0.45)
WBC (×10³/µL)	7.85 (6.61–10.15)	7.67 (6.32–8.99)	0.027	+0.14	+0.52 (+0.04 to +1.01)
Neutrophils (×10³/µL)	5.03 (3.90–6.59)	4.26 (3.51–5.41)	<0.001	+0.24	+0.70 (+0.36 to +1.03)
Lymphocytes (×10³/µL)	2.08 (1.50–2.54)	2.35 (1.91–2.85)	<0.001	−0.25	−0.33 (−0.49 to −0.14)
Monocytes (×10³/µL)	0.59 (0.47–0.72)	0.54 (0.45–0.65)	0.059	+0.12	+0.04 (−0.01 to +0.07)
Fibrinogen (mg/dL)	367.00 (310.00–421.00)	325.00 (276.75–371.00)	<0.001	+0.32	+43.00 (+24.48 to +60.00)
CRP (mg/L)	10.85 (3.65–24.18)	1.70 (0.70–4.83)	<0.001	+0.61	+7.00 (+3.80 to +11.55)
Albumin (g/L)	42.00 (39.25–44.75)	46.00 (44.00–48.00)	<0.001	−0.54	−4.00 (−5.00 to −2.00)
ALP (U/L)	76.00 (60.00–91.25)	66.00 (51.00–79.50)	<0.001	+0.27	+10.00 (+5.00 to +16.00)
LDH (U/L)	187.00 (168.00–218.00)	172.00 (155.50–196.00)	<0.001	+0.22	+14.00 (+6.00 to +23.00)
CEA (ng/mL)	2.58 (1.25–5.37)	1.41 (0.93–2.06)	<0.001	+0.38	+0.99 (+0.54 to +1.58)
CA-125 (U/mL)	16.20 (9.61–28.20)	11.90 (8.46–16.85)	<0.001	+0.28	+3.91 (+1.85 to +6.33)
CA-15-3 (U/mL)	17.80 (12.40–26.40)	16.00 (12.25–19.70)	0.004	+0.19	+2.50 (+0.60 to +4.50)
CA-19-9 (U/mL)	11.25 (6.01–21.05)	8.03 (4.85–15.65)	0.004	+0.19	+2.68 (+0.90 to +5.07)
NLR	2.48 (1.81–3.75)	1.85 (1.49–2.38)	<0.001	+0.38	+0.62 (+0.37 to +0.89)
MLR	0.29 (0.22–0.37)	0.23 (0.19–0.28)	<0.001	+0.34	+0.06 (+0.03 to +0.08)
LMR	3.48 (2.70–4.50)	4.36 (3.54–5.35)	<0.001	−0.34	−0.85 (−1.13 to −0.59)

Continuous variables were compared with the Mann-Whitney U test. IQR, interquartile range. Cliff's δ is a nonparametric effect size (absolute values of approximately 0.11, 0.28, and 0.43 correspond to small, medium, and large effects); positive values indicate higher values in the cancer group. The Hodges-Lehmann difference is the median of all pairwise cancer-minus-CIN differences, reported with a bootstrap 95% confidence interval (1000 resamples). Platelet count was not recorded in the cervical cancer cohort; consequently, platelet count and the platelet-derived indices PLR, SII, and AISI could not be computed for that group and are not included in this comparison. SIRI, although leukocyte-based, was not part of the prespecified index set (NLR, MLR, LMR) and was evaluated post hoc in response to peer review (main text, Section 3.5).

Table S2. Availability and missingness of key variables, by group.

Variable	Cancer, n available (missing %)	CIN, n available (missing %)
Age	137 (0.0)	238 (0.0)
Menopausal status	136 (0.7)	236 (0.8)
Hemoglobin	134 (2.2)	237 (0.4)
Neutrophils	134 (2.2)	237 (0.4)
Lymphocytes	134 (2.2)	237 (0.4)
CEA	113 (17.5)	226 (5.0)
CA-125	117 (14.6)	231 (2.9)
Fibrinogen	100 (27.0)	220 (7.6)
LDH	120 (12.4)	223 (6.3)
CRP	56 (59.1)	66 (72.3)
Albumin	50 (63.5)	62 (73.9)

Table S3. Dichotomized approximation of the prediction model: independent predictors of cervical cancer (complete-case n = 339).

Variable	OR	95% CI	p
Age ≥ 44 years	8.08	4.39–14.90	<0.001
CEA > 2.4 ng/mL	2.95	1.65–5.27	<0.001
NLR > 2.88	3.81	1.84–7.87	<0.001
Hemoglobin < 11.3 g/dL	2.50	1.25–4.98	0.009

Model equation: logit(P) = −2.79 + 2.09 × (age ≥ 44) + 1.08 × (CEA > 2.4) + 1.34 × (NLR > 2.88) + 0.91 × (hemoglobin < 11.3); AUC 0.831 (95% CI 0.783–0.878). Cutoffs are data-derived (Youden's J) and require external validation before any clinical application. OR, odds ratio; CI, confidence interval.

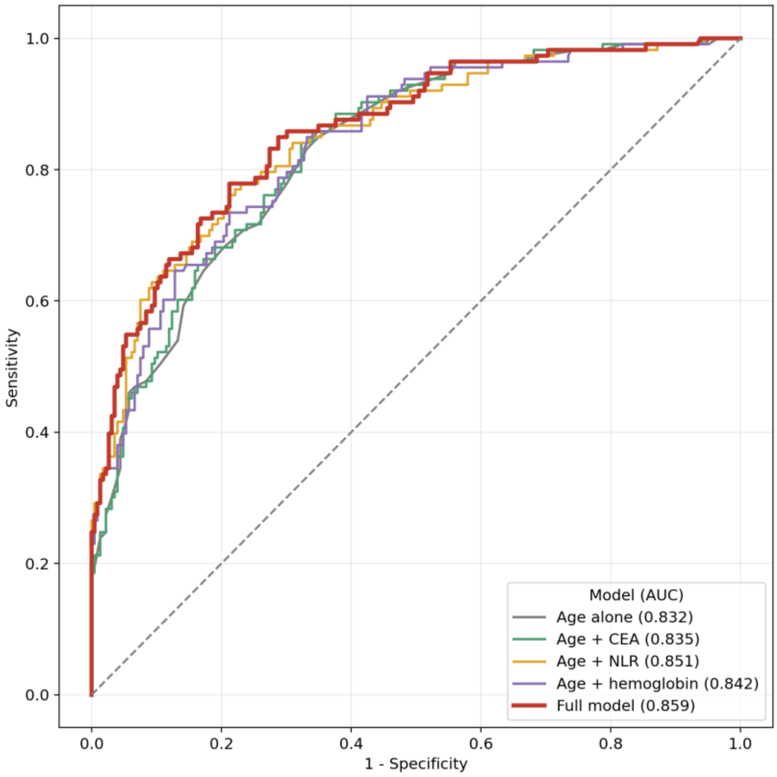

Figure S1. ROC curves for nested logistic models, illustrating the incremental value of CEA, NLR, and hemoglobin over age alone.

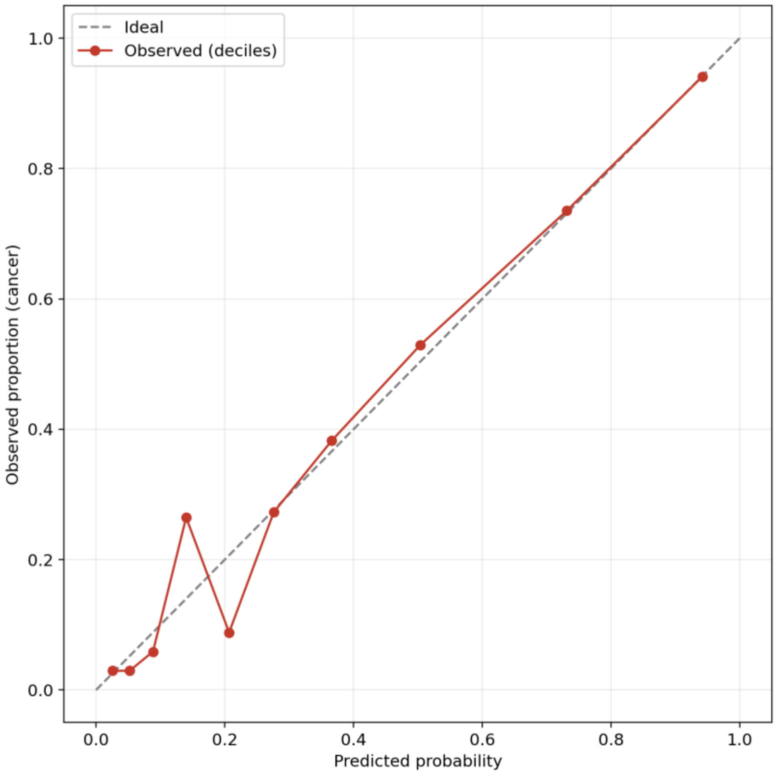

Figure S2. Calibration plot of the continuous four-variable model (decile observed vs. predicted probability). Calibration-in-the-large intercept 0.00; optimism-corrected calibration slope 0.962; Brier score 0.136.

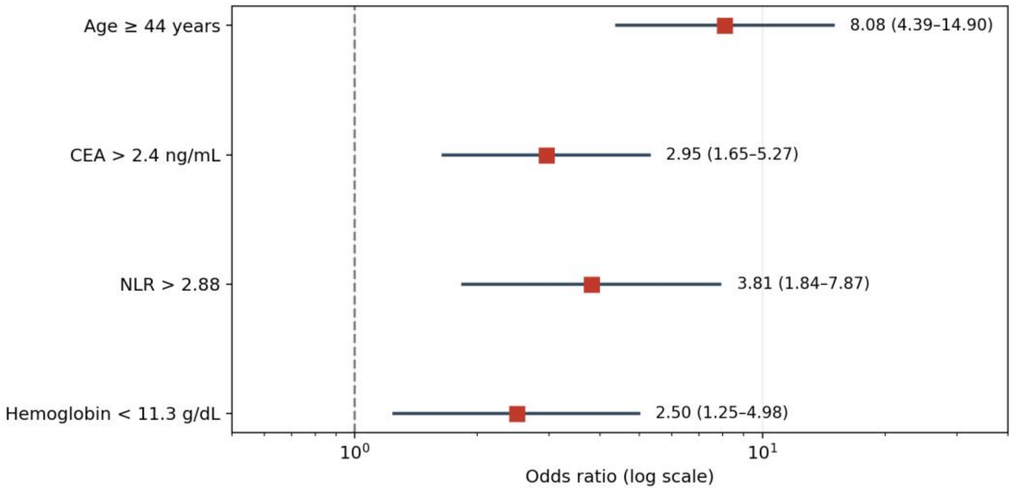

Figure S3. Forest plot of independent predictors of cervical cancer (multivariable logistic regression; odds ratios with 95% confidence intervals, log scale).

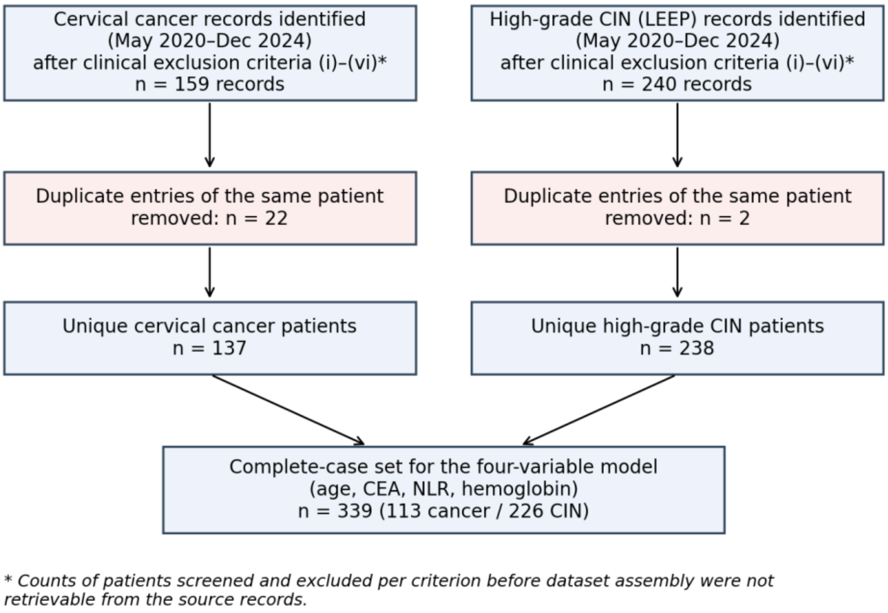

Figure S4. Participant flow. Counts of patients screened and excluded per clinical criterion before dataset assembly were not retrievable from the source records; duplicate removal and complete-case counts are derived from the assembled datasets.
