# Supplementary material for: Routine Blood-Based Parameters Associated with Invasive Cervical Cancer Versus High-Grade Cervical Intraepithelial Neoplasia: Development of a Retrospective Diagnostic Prediction Model
Source: Diagnostics (Basel). 2026 Sep 8;16(18):2884. doi: 10.3390/diagnostics16182884 (PMC13605862; doi:10.3390/diagnostics16182884)
Supplement: Supplementary file 1 [file diagnostics-16-02884-s001.zip › File S1. TRIPOD_Checklist_FINAL.pdf]

# TRIPOD 2015 Checklist

Manuscript: “Routine Blood-Based Parameters Associated with Invasive Cervical Cancer versus High-Grade Cervical Intraepithelial Neoplasia: Development of a Retrospective Diagnostic Prediction Model.” This study concerns model development with internal validation; items specific to external validation (10c, 10e, 12, 13c, 17, 19a) are marked not applicable. Section references point to the corresponding location in the submitted manuscript.

| Section / Item | Checklist item | Reported (section / element) |
|---|---|---|
| **Title and Abstract** | | |
| 1 | Identify the study as developing and/or validating a multivariable prediction model, the target population, and the outcome to be predicted. | Title |
| 2 | Provide a summary of objectives, study design, setting, participants, sample size, predictors, outcome, statistical analysis, results, and conclusions. | Abstract |
| **Introduction** | | |
| 3a | Explain the medical context and rationale for developing or validating the model, including references to existing models. | Introduction, paragraphs 1–3 |
| 3b | Specify the objectives, including whether the study describes development or validation of the model or both. | Introduction, final paragraph (development with internal validation) |
| **Methods** | | |
| 4a | Describe the study design or source of data, separately for development and validation sets if applicable. | Methods 2.1 |
| 4b | Specify key study dates (start/end of accrual; end of follow-up if applicable). | Methods 2.1 (May 2020–December 2024) |
| 5a | Specify key elements of the study setting, including number and location of centres. | Methods 2.1 (single tertiary center, Istanbul) |
| 5b | Describe eligibility criteria for participants. | Methods 2.1 |
| 5c | Give details of treatments received, if relevant. | Not applicable (diagnosis-time analysis; pretreatment sampling) |
| 6a | Clearly define the outcome that is predicted, including how and when assessed. | Methods 2.1 (histopathological cancer vs. high-grade CIN) |
| 6b | Report any actions to blind assessment of the outcome. | Methods 2.1–2.2 (outcome from routine pathology, independent of laboratory data) |
| 7a | Clearly define all predictors used in developing the model, including how and when measured. | Methods 2.2, 2.4 (age, NLR, CEA, hemoglobin; pretreatment) |
| 7b | Report any actions to blind assessment of predictors. | Methods 2.2 (automated laboratory platforms; retrospective extraction) |
| 8 | Explain how the study size was arrived at. | Methods 2.3 (all eligible consecutive patients; precision via 95% CIs) |
| 9 | Describe how missing data were handled, with details of any imputation. | Methods 2.4; Results 3.2; Supplementary Table S2 (complete-case primary; multiple-imputation sensitivity analysis; missingness reported) |
| 10a | Describe how predictors were handled in the analyses. | Methods 2.4 (continuous primary model; dichotomized clinical approximation) |
| 10b | Specify type of model, model-building procedures, and method of internal validation. | Methods 2.4 (logistic regression; bootstrap optimism correction, 1000 resamples) |
| 10c | For validation, describe how predictions were calculated. | Not applicable (model development with internal validation; no separate validation cohort) |
| 10d | Specify all measures used to assess model performance and to compare models. | Methods 2.4 (AUC, DeLong, bootstrap ΔAUC, calibration slope/intercept, Brier, DCA) |
| 10e | Describe any model updating. | Not applicable (model development with internal validation; no separate validation cohort) |
| 11 | Provide details on how risk groups were created, if done. | Methods 2.4 (dichotomized predictors at ROC-derived cutoffs) |

| Section / Item | Checklist item | Reported (section / element) |
|---|---|---|
| 12 | For validation, identify any differences from the development data. | Not applicable (model development with internal validation; no separate validation cohort) |
| **Results** | | |
| 13a | Describe the flow of participants, including numbers with and without the outcome. | Results 3.1–3.2 (137 cancer, 238 CIN after duplicate removal; complete-case n = 339) |
| 13b | Describe participant characteristics, including the number with missing data for predictors and outcome. | Results 3.1–3.2; Table 1, Supplementary Table S2 |
| 13c | For validation, compare the distribution of important variables with the development data. | Not applicable (model development with internal validation; no separate validation cohort) |
| 14a | Specify the number of participants and outcome events in each analysis. | Results 3.4–3.6; Table 3 (n per parameter); Tables 4–5; Supplementary Table S3 |
| 14b | Report the unadjusted association between each candidate predictor and outcome. | Results 3.3; Table 3; Supplementary Table S1 |
| 15a | Present the full prediction model (all regression coefficients and the intercept). | Results 3.4 (continuous model equation); Supplementary Table S3 (dichotomized approximation) |
| 15b | Explain how to use the prediction model. | Results 3.4 (continuous logit equation with probability transformation $P = 1/[1 + \exp(-\text{logit})]$); Supplementary Table S3 (dichotomized approximation) |
| 16 | Report performance measures (with CIs) for the model. | Results 3.4–3.5; Table 4; Supplementary Table S3; Figure 2; Supplementary Figures S1, S2 |
| 17 | If done, report the results from any model updating. | Not applicable (model development with internal validation; no separate validation cohort) |
| **Discussion** | | |
| 18 | Discuss any limitations of the study. | Discussion 4.4 (Limitations; PROBAST perspective) |
| 19a | For validation, discuss the results with reference to performance in the development data. | Not applicable (model development with internal validation; no separate validation cohort) |
| 19b | Give an overall interpretation of the results, considering objectives, limitations, and other evidence. | Discussion 4.1–4.2 |
| 20 | Discuss the potential clinical use of the model and implications for future research. | Discussion 4.3, 4.5; Conclusions |
| **Other information** | | |
| 21 | Provide information about the availability of supplementary resources and data. | Supplementary Table S1; Data Availability Statement |
| 22 | Give the source of funding and the role of the funders. | Funding statement (no external funding) |
