# Supplementary material for: Routine Blood-Based Parameters Associated with Invasive Cervical Cancer Versus High-Grade Cervical Intraepithelial Neoplasia: Development of a Retrospective Diagnostic Prediction Model
Source: Diagnostics (Basel). 2026 Sep 8;16(18):2884. doi: 10.3390/diagnostics16182884 (PMC13605862; doi:10.3390/diagnostics16182884)
Supplement: Supplementary file 1 [file diagnostics-16-02884-s001.zip › File S2. STARD_Checklist_FINAL.pdf]

STARD 2015 Checklist

Manuscript: “Routine Blood-Based Parameters Associated with Invasive Cervical Cancer versus High-Grade Cervical Intraepithelial Neoplasia: Development of a Retrospective Diagnostic Prediction Model.” Section references point to the corresponding location in the submitted manuscript.

Section / Item	Checklist item	Reported (section / element)
**Title or abstract**		
1	Identification as a study of diagnostic accuracy using at least one measure of accuracy (e.g., sensitivity, specificity, predictive values, AUC).	Title; Abstract (AUC)
**Abstract**		
2	Structured summary of study design, methods, results, and conclusions.	Abstract (unstructured per journal requirements; includes study design, methods, results, and conclusions)
**Introduction**		
3	Scientific and clinical background, including the intended use and clinical role of the index test.	Introduction, paragraphs 1–3
4	Study objectives and hypotheses.	Introduction, final paragraph
**Methods**		
5	Whether data collection was planned before (prospective) or after (retrospective) the index test and reference standard.	Methods 2.1 (retrospective diagnostic-accuracy study with two-gate (case-control) sampling)
6	Eligibility criteria.	Methods 2.1 (inclusion/exclusion)
7	On what basis potentially eligible participants were identified.	Methods 2.1 (histopathology-confirmed cancer; LEEP-confirmed CIN)
8	Where and when potentially eligible participants were identified (setting, location, dates).	Methods 2.1 (single center; May 2020–December 2024)
9	Whether participants formed a consecutive, random, or convenience series.	Methods 2.1 (consecutive)
10a	Index test, in sufficient detail to allow replication.	Methods 2.2, 2.4 (laboratory parameters; model and cutoffs)
10b	Reference standard, in sufficient detail to allow replication.	Methods 2.1, 2.2 (histopathology / LEEP grade)
11	Rationale for choosing the reference standard.	Introduction; Methods 2.1 (histopathology is the diagnostic gold standard)
12a	Definition of and rationale for index-test cutoffs, distinguishing prespecified from exploratory.	Methods 2.3, 2.4 (Youden-derived cutoffs; apparent performance noted)
12b	Definition of and rationale for reference-standard categories.	Methods 2.1 (invasive carcinoma vs. CIN 2/CIN 3/CIN 2-3)
13a	Whether clinical information and reference-standard results were available to readers of the index test.	Methods 2.2 (laboratory values from automated platforms; retrospective extraction)
13b	Whether clinical information and index-test results were available to assessors of the reference standard.	Methods 2.1–2.2 (routine histopathology, independent of laboratory analysis)
14	Methods for estimating or comparing measures of diagnostic accuracy.	Methods 2.3, 2.4 (ROC, DeLong, bootstrap ΔAUC, calibration, DCA)
15	How indeterminate index-test or reference-standard results were handled.	Methods 2.1 (cases lacking baseline laboratory data excluded)
16	How missing data on index test and reference standard were handled.	Methods 2.4; Results 3.2; Supplementary Table S2 (complete-case primary; multiple-imputation sensitivity analysis; missingness reported)
17	Any analyses of variability in diagnostic accuracy, distinguishing prespecified from exploratory.	Methods 2.4; Results 3.6 (age-matched and subgroup analyses; exploratory)
18	Intended sample size and how it was determined.	Methods 2.3 (all eligible consecutive patients; no a priori sample-size calculation)
**Results**		

Section / Item	Checklist item	Reported (section / element)
19	Flow of participants, using a diagram.	Results 3.1; Supplementary Figure S4 (flow diagram; pre-assembly screening counts not retrievable)
20	Baseline demographic and clinical characteristics of participants.	Results 3.1; Table 1
21a	Distribution of severity of disease in those with the target condition.	Results 3.1; Table 1 (FIGO stage, histology)
21b	Distribution of alternative diagnoses in those without the target condition.	Results 3.1; Table 1 (CIN 2/CIN 3/CIN 2-3)
22	Time interval and any clinical interventions between index test and reference standard.	Methods 2.2 (blood drawn at diagnosis, before treatment)
23	Cross tabulation (or distribution) of index-test results by the reference standard.	Results 3.2–3.4; Table 2, Table 3; Figure 1
24	Estimates of diagnostic accuracy and their precision (e.g., 95% CIs).	Results 3.3–3.5; Tables 3–4 and Supplementary Table S3 (AUCs with 95% CIs)
25	Any adverse events from performing the index test or reference standard.	Not applicable (routine blood test and standard histopathology)
**Discussion**		
26	Study limitations, including sources of bias, statistical uncertainty, and generalisability.	Discussion 4.4 (Limitations; PROBAST perspective)
27	Implications for practice, including intended use and clinical role of the index test.	Discussion 4.3, 4.5; Conclusions
**Other information**		
28	Registration number and name of registry.	Not applicable (retrospective study; not registered)
29	Where the full study protocol can be accessed.	Available from the corresponding author on request
30	Sources of funding and other support; role of funders.	Funding statement (no external funding)
